# Hepatokines and Non-Alcoholic Fatty Liver Disease: Linking Liver Pathophysiology to Metabolism

**DOI:** 10.3390/biomedicines9121903

**Published:** 2021-12-14

**Authors:** Tae Hyun Kim, Dong-Gyun Hong, Yoon Mee Yang

**Affiliations:** 1 Research Institute of Pharmaceutical Sciences, College of Pharmacy, Sookmyung Women’s University, Seoul 04310, Korea; thkim@sookmyung.ac.kr; 2 Department of Pharmacy, Kangwon National University, Chuncheon 24341, Korea; redcocuss96@kangwon.ac.kr; 3 KNU Researcher Training Program for Developing Anti-Viral Innovative Drugs, Kangwon National University, Chuncheon 24341, Korea

**Keywords:** ANGPTL, energy metabolism, Fetuin, FGF21, inter-organ communication

## Abstract

The liver plays a key role in maintaining energy homeostasis by sensing and responding to changes in nutrient status under various metabolic conditions. Recently highlighted as a major endocrine organ, the contribution of the liver to systemic glucose and lipid metabolism is primarily attributed to signaling crosstalk between multiple organs via hepatic hormones, cytokines, and hepatokines. Hepatokines are hormone-like proteins secreted by hepatocytes, and a number of these have been associated with extra-hepatic metabolic regulation. Mounting evidence has revealed that the secretory profiles of hepatokines are significantly altered in non-alcoholic fatty liver disease (NAFLD), the most common hepatic manifestation, which frequently precedes other metabolic disorders, including insulin resistance and type 2 diabetes. Therefore, deciphering the mechanism of hepatokine-mediated inter-organ communication is essential for understanding the complex metabolic network between tissues, as well as for the identification of novel diagnostic and/or therapeutic targets in metabolic disease. In this review, we describe the hepatokine-driven inter-organ crosstalk in the context of liver pathophysiology, with a particular focus on NAFLD progression. Moreover, we summarize key hepatokines and their molecular mechanisms of metabolic control in non-hepatic tissues, discussing their potential as novel biomarkers and therapeutic targets in the treatment of metabolic diseases.

## 1. Introduction

The global prevalence of obesity has been increasing over several decades, reaching epidemic levels, and thus, raising serious public health concerns [1]. Obesity greatly increases the risk of metabolic syndrome, including insulin resistance, type 2 diabetes, fatty liver disease, cardiovascular disease, and neurodegenerative conditions, thereby significantly contributing to greater morbidity and mortality [1]. Moreover, obesity-induced chronic inflammation, particularly in the liver, adipose tissue, and skeletal muscle, plays a crucial role in the development of local and systemic insulin resistance, simultaneously inducing a range of metabolic disturbances in multiple organs via inter-organ crosstalk [2,3]. Given the concurrent detrimental influence of obesity on various organs, obesity-driven metabolic disorders are largely attributed to dysregulated multi-directional interactions between organ metabolism. In other words, inter-organ communication via autocrine, paracrine, and endocrine signals regulates systemic energy homeostasis, which can be disturbed by a disequilibrium between energy intake and expenditure (e.g., obesity, hepatic steatosis, etc.).

Non-alcoholic fatty liver disease (NAFLD) refers to the spectrum of chronic liver disease progression in the absence of excessive alcohol consumption, ranging from simple hepatic steatosis to severe pathological conditions, including non-alcoholic steatohepatitis (NASH), fibrosis, cirrhosis, and hepatocellular carcinoma [4,5,6,7]. Hepatic steatosis is characterized by the accumulation of ectopic lipids in hepatocytes by more than 5% of total liver mass, exhibiting mostly benign and/or mild clinical symptoms. However, this becomes problematic after developing NASH with the signs of hepatocellular damage, inflammation and fibrotic changes, favoring its progression towards more debilitating conditions. In parallel, emerging evidence has also revealed that patients with NAFLD have greater susceptibility to various infectious diseases, including bacterial pneumonia, *Helicobacter pylori* infection, urinary tract infection, *Clostridium difficile* colitis, and coronavirus disease 2019 (COVID-19) in close association with low-grade chronic inflammation, impaired innate immunity, and/or vitamin D deficiency [8,9]. NAFLD serves as an early hepatic manifestation that is primarily responsible for the progression of a variety of metabolic disorders [4,5,6,7], implying the multifactorial features of NAFLD and its close link with other comorbidities. Given the heterogenous pathogenesis of NAFLD and its increasing prevalence, leading international societies of hepatology have recently proposed new nomenclature ‘metabolic dysfunction-associated fatty liver disease’ (MAFLD) in the replacement of NAFLD, emphasizing more on its function in metabolic dysregulation, in order to better reflect the current knowledge, as well as its diverse etiologies of metabolic liver disease [10,11].

Recent advances in comprehensive genetic, transcriptomic, and proteomic technologies have provided insights into the role of the liver as an endocrine organ central to metabolism. Moreover, growing evidence has revealed that the liver mediates metabolic regulation through the release of various secretory factors, including hepatokines [12,13,14,15]. Hepatokines are hormone-like proteins primarily secreted by hepatocytes, with the hepatokine secretory profile known to be markedly disturbed in NAFLD. In fact, NAFLD frequently precedes dysfunction in other organs during the pathogenesis of systemic metabolic diseases. Therefore, it is highly likely that altered hepatokine secretion at NAFLD onset will significantly impair the inter-organ signaling crosstalk, which may trigger the progression of complex and multifaceted metabolic dysregulation. In this review, we first outline distinctive roles of the liver as a hepatokine-producing organ under normal physiology. Then, we discuss alterations in hepatokine secretion during NAFLD development and their impact on metabolic disease. The current review also summarizes recent discoveries regarding the molecular mechanisms and effects of key hepatokines, in addition to their potential as novel diagnostic and therapeutic targets in metabolic disease treatment.

## 2. Role of the Liver in Metabolism under Normal Physiology

The liver contributes to the maintenance of systemic metabolism by controlling complex pathways that coordinate nutrient intake and energy expenditure. Blood flows into the liver from the heart through the hepatic artery (~25% of total blood volume) or from the gastrointestinal tract via the portal vein (~75%), with most of the nutrients (e.g., glucose and lipids) absorbed in the intestines which are delivered prior to their entry into systemic circulation. These anatomical and structural features enable the liver to sense and promptly respond to changes in nutrient availability [16,17].

The liver serves as a key regulator of glucose metabolism by orchestrating hepatic glucose production and glycogen storage. In a postprandial state, hepatic glucose uptake is upregulated in response to elevated plasma glucose and insulin levels. Then, the absorbed glucose is stored in the liver as glycogen or is utilized for fatty acid synthesis (i.e., de novo lipogenesis). Under the influence of insulin secreted in response to elevated blood glucose, hepatic glucose production and glycogenolysis are suppressed in order to normalize blood glucose concentrations [13,14]. During the fasting state, the liver upregulates blood glucose levels by stimulating hepatic glucose production and glycogen breakdown via transcriptional and non-transcriptional mechanisms [13,18,19], thus increasing the supply of glucose as a major energy source to non-hepatic peripheral tissues (e.g., brain, adipose tissue, skeletal muscle). The liver also plays a key role in lipid metabolism in response to nutrient availability, as well as insulin. Following a meal, insulin facilitates hepatic fatty acid uptake, the synthesis and storage of triglycerides (TG) via the utilization of dietary fatty acids, and promotes de novo lipogenesis in the liver [20], favoring long-term lipid storage. However, under fasting, wherein glucose availability is low, the liver carries out lipid oxidation and acutely produces ketone bodies, which serve as an alternative fuel source for non-hepatic tissues in order to meet energy demands [20,21]. As the liver encounters repetitive fasting-feeding transitions throughout the life cycle of living organisms, the hepatic regulation of metabolism, including energy production, expenditure, storage, and redistribution, is critical for maintaining systemic energy homeostasis under normal physiology.

## 3. Overview of Hepatokines

The liver regulates physiology and metabolism via the production and secretion of various plasma proteins, including albumin, coagulation factors, complement factors, transport proteins, and other factors [14]. Although the liver has long been recognized as a secretory organ, recent advances in mass spectrometry-based quantitative proteomics have enabled researchers to identify ~10,200 proteins produced in the human liver [22], with up to 40% of hepatic transcripts encoding secretory proteins [23]. Similarly, quantitative analysis of the mouse liver and plasma proteome identified 7099 and 4727 proteins, respectively, with ~25% of these overlapping with plasma proteins, suggestive of their secretion [12,24]. As mentioned above, the liver is directly connected to systemic circulation, receiving a substantial amount of blood from the heart and gastrointestinal tract, exchanging nutrients and other substances within the sinusoids, and then draining blood via the hepatic vein and inferior vena cava towards the heart for recirculation throughout the body [14]. Considering its secretory capacity as an endocrine organ, it is plausible to expect that the liver plays a fundamental role in inter-organ crosstalk through the release of secretory proteins, including hepatokines.

Analogous to adipose tissue-derived adipokines and skeletal muscle-derived myokines, hepatokines are a class of organokines, meaning a group of secretory proteins that are exclusively produced by the parenchymal cell type of respective tissue. The significance of hepatokines in the regulation of various biological processes in autocrine, paracrine, and endocrine fashion has been recently highlighted [13,25]. Given the functional features of hepatocytes, which constitute ~80% of the volume and ~70% of the total cell number within the whole liver, several studies have shown that normal mouse hepatocytes release more than 500 secretory proteins, which may or may not contain an N-terminal secretory peptide [12,26]. Since emerging evidence has demonstrated that factors secreted from hepatocytes actively mediate metabolic regulation between the liver and other organs, hepatokines have drawn increasing attention due to their capacity for metabolic regulation, making them novel targets for the modulation of energy homeostasis and the treatment of metabolic disorders [12,13,14,15]. In this review, we summarized target organs or cells of important hepatokines and their biological functions (Table 1).

## 4. Hepatokines and NAFLD

It has been established that NAFLD is strongly associated with other metabolic comorbidities, including obesity, type 2 diabetes, dyslipidemia, cardiovascular disease, colonic diverticulosis, and neurodegenerative conditions [1,4,5,6,7,14,91]. Hepatic steatosis, which refers to a pathological state of the liver characterized by an accumulation of lipid content at over ~5% of the total organ weight, is closely associated with insulin resistance in multiple organs, as supported by several studies demonstrating impaired insulin action in both lean and non-diabetic obese individuals [14,92,93,94]. Moreover, hepatic steatosis usually develops prior to the accumulation of lipid in skeletal muscle, macrophage-driven inflammation, extrahepatic insulin resistance, and hyperglycemia [14,16,95], suggestive of its potential as an early indicator of systemic metabolic dysregulation. Interestingly, similar to adipose tissue or skeletal muscle where the secretion of respective organokines is altered via overnutrition, hepatic gene expression and protein content are also regulated in response to caloric overload, and these changes have been revealed to be strongly associated with the onset of insulin resistance and type 2 diabetes [26,96,97,98]. Many of the liver-secreted proteins that are upregulated in the plasma of subjects with type 2 diabetes were capable of inducing insulin resistance, supportive of the pathophysiological role of hepatokines in metabolic dysregulation [99]. Recent studies have provided valuable insight into the impact of ectopic fat accumulation in hepatocytes on alterations in the hepatic proteome, with suppressed protein synthesis observed in the livers of obese mice [100]. Furthermore, metabolic remodeling was mediated via changes in the translation/secretion processes or post-translational modifications, with minor changes at the transcriptional level [12,26]. In line with this notion, approximately 20% of proteins with an N-terminal signal peptide were differentially secreted from mouse steatotic hepatocytes compared to normal hepatocytes, and some of these proteins were found to induce insulin resistance and pro-inflammatory signaling [26]. The above-described findings indicate that a substantial fraction of the hepatic proteome is heavily distributed during NAFLD progression, which may significantly impair energy homeostasis and systemic metabolism. However, relatively a small number of hepatokines have been identified, with a few of their metabolic functions and related regulatory mechanism(s) in NAFLD progression investigated. Considering the close association between NAFLD and hepatokines in the context of metabolic disease progression, we have summarized recent findings on the function of some key hepatokines in the regulation of metabolism via organ-to-organ crosstalk (Table 1).

### 4.1. Angiopoietin-Like Proteins (ANGPTLs)

**Figure 1:**
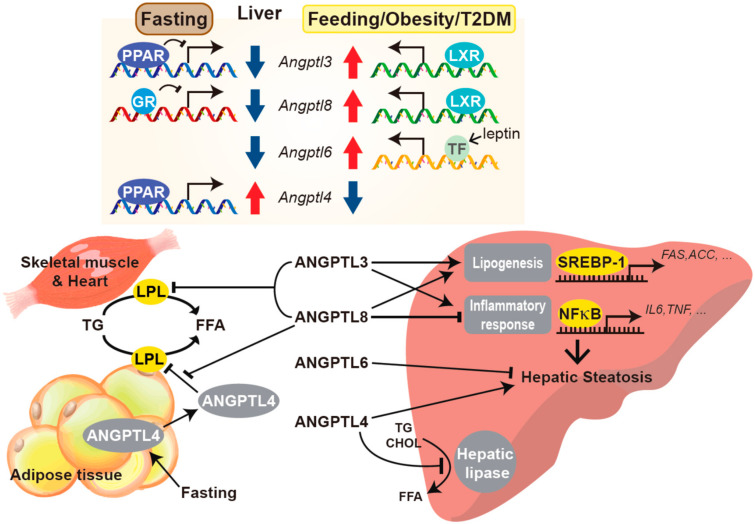
The role of ANGPTL family proteins in lipid metabolism in the liver and non-hepatic tissues. ANGPTL family proteins are hepatokines primarily produced and secreted by the liver, regulating various cellular processes, including lipid metabolism, within as well as outside the liver. The upper panel presents the transcription factors that regulate the gene expression of ANGPTL members under fasting or feeding/obesity/type 2 diabetes (T2DM) conditions. The transcription of ANGPTL3, ANGPTL8, and ANGPTL6 is upregulated by liver-X-receptor (LXR) activation or leptin signaling following feeding or under metabolic disorders, such as obesity and type 2 diabetes mellitus (T2DM), while peroxisome proliferator-activated receptor (PPAR) and glucocorticoid receptor (GR) suppress the transactivation of *ANGPTL3* and *ANGPTL8* under fasting conditions, respectively. Circulating ANGPTL proteins then serve as lipoprotein lipase (LPL) inhibitors in non-hepatic tissues, while liver-derived ANGPTL4 inhibits hepatic lipase, resulting in decreased triglyceride clearance. Therefore, ANGPTL proteins contribute to the development of hepatic steatosis by regulating lipogenesis and/or the inflammatory response, as depicted above. ACC: Acetyl-CoA carboxylase; CHOL: Cholesterol; FAS: Fatty acid synthase; FFA: Free fatty acid; IL6: Interleukin-6; NF-ĸB: Nuclear factor kappa-light-chain-enhancer of activated B cells; SREBP: Sterol regulatory-element binding protein; TF: Transcription factor; TG: Triglyceride; TNF: Tumor necrosis factor.

ANGPTLs are a family of glycoproteins primarily secreted from the liver, and all of these share an N-terminal coiled-coil domain and a C-terminal fibrinogen-like domain, except for ANGPTL8, which lacks the latter [40,44,101]. To date, eight ANGPTLs have been identified (ANGPTL1–ANGPTL8), all of which are capable of regulating angiogenesis as angiopoietins [15,27,40,101,102,103,104]. Despite their structural similarity, ANGPTLs were originally regarded as orphan ligands since they do not bind to classical angiopoietin cognate receptors, including tyrosine kinase with immunoglobulin-like and EGF-like domain 1 (Tie1) and TEK receptor tyrosine kinase (Tie2) [101,105,106]. Thereafter, human leukocyte immunoglobulin-like receptor B2 and its mouse orthologue paired immunoglobulin-like receptor were defined as the receptors for ANGPTL1, 2, 5, and 7 [107]. Angiopoietin-like proteins play an important role in lipid metabolism, with ANGPTL3, 4, 6, and 8 involved in lipoprotein metabolism and modulating plasma lipid levels via the regulation of lipoprotein lipase (LPL) and endothelial lipase-dependent TG hydrolysis (Figure 1) [15,27,40,101].

ANGPTL3 is predominantly synthesized and secreted by the liver, while ANGPTL4 and ANGPTL8 are also expressed in other tissues, such as adipose tissue [28]. Activated after feeding, ANGPTL3 inhibits LPL through direct binding via the N-terminal coiled-coil domain, subsequently dissociating active LPL dimers into inactive monomers with the help of ANGPTL8 [15,27,28,29,30], which leads to the increased storage of lipoprotein-derived fatty acids in white adipose tissue [31]. In addition, ANGPTL3 inhibition decreases plasma TG and free fatty acid levels in mice [28], while also reducing VLDL size and lipid content in an endothelial lipase-dependent manner [15,32]. Interestingly, fasting conditions as well as very low-calorie intake markedly decreased serum ANGPTL3 in obese individuals, while ANGPTL4 showed the opposite or a distinct trend [108]. Furthermore, the circulating levels of both may be independent risk factors and potential predictors of coronary atherosclerosis [109]. Recent studies have provided insight into the metabolic role of circulating ANGPTL3 in preclinical models, wherein the treatment with an anti-sense oligonucleotide targeting *Angptl3* mRNA in low-density lipoprotein receptor knockout mice resulted in a dramatic reduction of liver TG content, as well as atherosclerosis progression [110]. The ob/ob mice, vaccinated with a peptide targeting ANGPTL3, also exhibited a marked decrease in diet-induced obesity and hepatic steatosis [111], suggestive of the metabolic impact of ANGPTL3 on NAFLD. In addition, the treatment with evinacumab alone or in combination with other lipid-lowering medications, such as statins and PCSK9 inhibitors, showed promising results for ameliorating hyperlipidemia in comparison to general lipid-lowering drugs alone in a phase 3 clinical trial, as well as in rodent models [112,113,114,115]. These findings indicate that hepatokine ANGPTL3 acts as an important regulator of lipoprotein metabolism and has great potential for the treatment of atherosclerosis [28].

ANGPTL4 is the most extensively studied member of the ANGPTL family and is primarily produced from several organs, including adipose tissue and the liver [116,117]. In humans, ANGPTL4 is predominantly expressed in the liver, followed by adipose tissue, while in mice the highest levels of ANGPTL4 are observed in white and brown adipose tissue [118]. ANGPTL4 was originally referred to as fasting-induced adipose factor, with its expression increasing in multiple tissues under fasting or hypoxia [34]. Similarly, *Angptl4* transcript levels in the liver are upregulated upon fasting via peroxisome proliferator-activated receptor α (PPARα) signaling and are suppressed after refeeding [15,119]. ANGPTL4 plays a crucial role in various biological and pathophysiological processes, including the maintenance of energy homeostasis, TG metabolism, wound repair, angiogenesis, tumorigenesis, etc. [15,27,34,104,120]. The full-length ANGPTL4 undergoes proteolytic cleavage at the linker region into its N-terminal and C-terminal portions, which exhibit distinct biological functions [34]. Emerging evidence has demonstrated that both full-length ANGPTL4 and N-terminal ANGPTL4 upregulate plasma TG levels via the inhibition of LPL activity and the suppression of TG-containing lipoprotein clearance from plasma [15,34,35,36,37]. Meanwhile, C-terminal ANGPTL4 not only increases adipocyte lipolysis [38], but also regulates several processes beyond lipid metabolism, including the metabolic flexibility of metastatic cancer cells, wound healing in the skin, and pulmonary inflammation [121,122,123]. Given the therapeutic potential of ANGPTL4 in metabolic disorders, a number of studies have sought to validate the functional role of ANGPTL4 in energy metabolism using preclinical animal models, with global ANGPTL4 depletion via neutralizing monoclonal antibodies or germline *Angptl4* knockout mice and monkeys exhibiting severe metabolic abnormalities under high-fat diet (HFD) feeding [124,125,126]. In line with these findings, there have been conflicting observations regarding the levels of circulating ANGPTL4 in humans, with several studies reporting that ANGPTL4 plasma levels were elevated in patients with type 2 diabetes and obese non-diabetic subjects [108,127,128,129,130], while the concentration of ANGPTL4 was lower in another cohort of type 2 diabetes patients and showed an inverse correlation with blood glucose levels and the insulin resistance index [39]. These discrepancies highlight the necessity for a more fundamental approach towards understanding the regulatory mechanism of ANGPTL4 in biological and metabolic processes. Interestingly, several studies have found that ANGPTL4 distinctively regulates lipid and glucose metabolism in a tissue-dependent manner under various pathophysiological conditions. Adenovirus-driven *Angptl4* overexpression in mice improved systemic glucose tolerance and enhanced the insulin-induced suppression hepatic glucose production. However, mice developed hepatic steatosis and hyperlipidemia, including elevated plasma concentration of TGs, free fatty acids, total, and HDL cholesterol [39]. Transgenic mice with adipose tissue-specific *Angptl4* overexpression exhibited glucose intolerance and impaired insulin sensitivity in skeletal muscle, as well as aggravated hepatic steatosis when subjected to long-term HFD feeding [131]. Recent studies revealed that the genetic ablation of *Angptl4* in liver or adipose tissue, as well as targeted pharmacological inhibition of ANGPTL4 in hepatocytes significantly reduced circulating TG and cholesterol levels in mice, protecting them against diet-induced obesity, glucose intolerance, liver steatosis, and atherogenesis [124,132]. Taken together, all these findings strongly suggest a complex molecular mechanism of ANGPTL4 function in vivo, highlighting the need for further investigation into the tissue-dependent metabolic roles of ANGPTL4.

ANGPTL6, also referred to as angiopoietin-related growth factor, is another circulating protein predominantly secreted from the liver into the blood, with a key role in the maintenance of glucose and lipid metabolism [133,134]. Mice with genetic ablation of *Angptl6* exhibited greater obesity phenotypes, with increased hepatic fat accumulation and insulin resistance, in conjunction with impaired energy utilization [133], while *Angptl6* overexpression improved hepatic steatosis and obesity under increased energy expenditure [133]. In addition, several studies have demonstrated that the adenosine monophosphate-activated kinase (AMPK) pathway is activated by ANGPTL6, in parallel to enhanced insulin signaling in skeletal muscle [40]. Furthermore, ANGPTL6 inhibited hepatic gluconeogenesis by suppressing FOXO1 activity downstream of PI3K-Akt [41,42]. The treatment with recombinant ANGPTL6 increased mitochondrial oxygen consumption and PPARα expression via extracellular signal-regulated kinase (ERK)/mitogen-activated protein kinase signaling in cultured adipocytes [43]. Although there is limited information on the pathophysiological significance of ANGPTL6 up-/downregulation in humans under different metabolic statuses, emerging evidence has revealed that serum ANGPTL6 levels were increased in individuals suffering from type 2 diabetes, metabolic syndrome, and preeclampsia [135,136,137,138]. In particular, ANGPTL6 levels were upregulated in patients with obesity and type 2 diabetes, which are positively correlated with the fasting serum glucose concentration and preceding the development of representative metabolic syndrome symptoms, including dyslipidemia (low HDL and high plasma TG levels) [135,138]. Interestingly, mice subjected to HFD feeding exhibited increased serum leptin levels in conjunction with hepatic ANPTL6 expression, which were normalized via exercise training in both animals and humans [139]. In particular, the leptin treatment significantly increased hepatic ANGPTL6 expression at both the transcript and protein level in vitro and in vivo, indicating that ANGPTL6 is indeed directly regulated via leptin signaling in the context of metabolic disturbance [139]. These findings strongly support the therapeutic potential of ANGPTL6 against metabolic disorders, necessitating a more comprehensive approach for the elucidation of its molecular mechanism of action in the future.

ANGPTL8, also referred to as lipasin, betatrophin, and RIFL (refeeding induced in fat and liver), has recently been identified as the 8th member of the ANGPTL family, while possessing distinct structural features compared to the rest, as exemplified by the lack of a C-terminal fibrinogen/angiopoietin-like domain [40,44,101]. ANGPTL8 is mainly produced and secreted from the liver and adipose tissue in mice, as well as humans, although the main site of ANGPTL8 expression may vary among species [140]. ANGPTL8 also plays an important role in regulating LPL activity in concert with ANGPTL3 and ANGPTL4, presumably due to the high structural homology of its N-terminal domain with ANGPTL3 and ANGPTL4 [45]. Interestingly, accumulating evidence has demonstrated that ANGPTL8 does not operate alone with regards to LPL regulation, rather forming a complex with ANGPTL3 to elicit a maximal inhibitory effect on LPL activity [31,141]. Conversely, ANGPTL8 binds to ANGPTL4 and impairs its ability to inhibit LPL activity [141], thus acting as an endogenous inhibitor of ANGPTL4.

ANGPTL8 originally drew significant interest based on the initial findings regarding its possible roles in the induction of pancreatic β cell proliferation, where the term ‘betatrophin’ initially came from [44,142]. However, a series of subsequent studies deemed these findings irreproducible [142], reporting a marginal effect of ANGPTL8 on β cell proliferation [44,143,144,145]. Recent studies have provided greater insight into the role of ANGPTL8 in other metabolic processes, with adenovirus-mediated *Angptl8* overexpression reducing fasting blood glucose levels and improving glucose tolerance, as well as insulin sensitivity in diabetic mice via the suppression of hepatic gluconeogenic gene expression downstream of Akt [146]. ANGPTL8 has been reported to negatively regulate NF-ĸB, a critical driver of inflammatory signaling, by triggering the autophagic degradation of IKKγ, an inhibitory regulator of IĸBα [44,147]. Of note, the treatment with *Angptl8* anti-sense oligonucleotides markedly alleviated HFD-induced hepatic steatosis in rodents [148], presumably due to ANGPTL8 promoting lipogenesis via upregulating nuclear sterol-regulatory element binding protein 1 (SREBP1) [149]. Moreover, the treatment of 3T3-L1 cells or HepG2 cells with recombinant ANGPTL8 resulted in a significant suppression of lipolysis, as indicated by the decreased expression of adipose TG lipase and hormone-sensitive lipase through Akt and mTOR activation [44,46]. Furthermore, the overexpression of ANPTGL8 in the liver improved insulin sensitivity and glucose tolerance, with enhanced Akt phosphorylation and improved insulin signaling in isolated primary hepatocytes [47].

Hepatic ANGPTL8 expression is upregulated in response to a number of nutritional stress conditions, including fasting/refeeding, hyperglycemia, hyperlipidemia, and endoplasmic reticulum stress, many of which are also well-established risk factors for NAFLD progression [44,48,150,151]. While accumulating evidence indicates the potential of ANGPTL8 as a novel biomarker and/or therapeutic target for a myriad of metabolic disorders, there have been conflicting results showing a bidirectional correlation of circulating ANGPTL8 levels with NAFLD, obesity, type 2 diabetes, and dyslipidemia [15,44,152,153,154,155,156], which altogether, warrant a further comprehensive investigation.

### 4.2. Fetuin-A and Fetuin-B

Fetuins are glycoproteins primarily synthesized from the liver and secreted into the bloodstream. Fetuins belong to the cystatin superfamily of proteins that mediate the transport of multiple cargo substances in the blood as carriers. While serum albumin is the representative and most abundant plasma carrier protein in adults, Fetuins are more enriched in fetal blood, which are identified as the major plasma proteins during fetal life, peaking in concentration shortly after birth, in a transient fashion [157]. Accumulating evidence has revealed that Fetuins, namely Fetuin-A and Fetuin-B, play crucial roles in a variety of cellular processes within the context of metabolic homeostasis.

**Figure 2:**
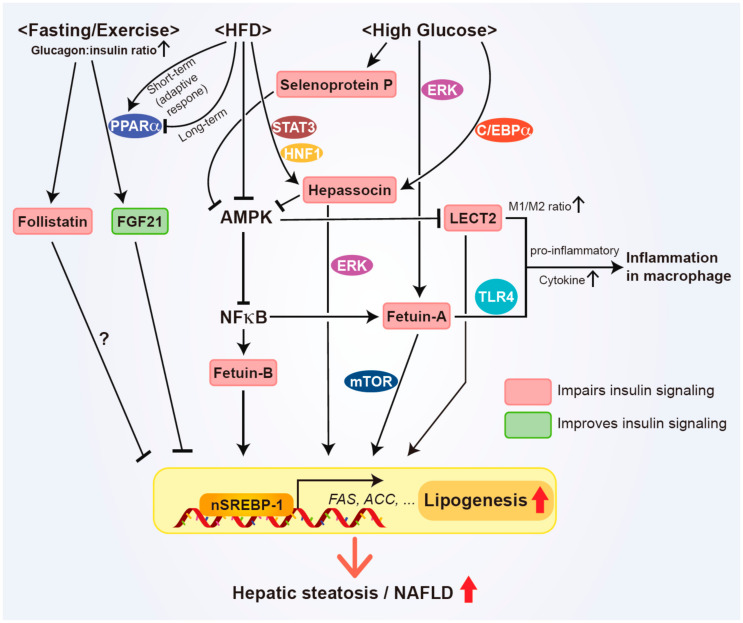
The impact of hepatokines on NAFLD progression and their associated signaling pathways. Most of the hepatokine expression is upregulated under HFD feeding and/or hyperglycemia. FGF21 has been reported to decrease or increase under HFD feeding. Hepatokines, except for FGF21, impair insulin signaling in hepatocytes, as well as non-hepatic tissues in an endocrine manner, with Fetuin-A and LECT2 promoting the macrophage inflammatory response by upregulating pro-inflammatory cytokine production. Fetuins, LECT2, and Hepassocin promote hepatic steatosis via the upregulation of lipogenic genes, whereas FGF21 suppressed it. The regulatory mechanism of follistatin on lipogenesis is unknown.

Fetuin-A, also known as alpha2-HS-glycoprotein, is the first hepatokine discovered to mediate organ-to-organ communication in the regulation of metabolic homeostasis [12,13,14,15]. Fetuin-A is predominantly produced and secreted from the liver, with recent studies describing other sources of circulating Fetuin-A, including visceral and subcutaneous adipose tissue [158,159,160,161]. Fetuin-A initially drew great attention as a natural inhibitor of the insulin receptor tyrosine kinase in the liver, adipose tissue, and skeletal muscle [13,49,50,51]. These findings were further supported by improved insulin signaling, decreased body fat, and protection from diet-induced body weight gain in Fetuin-A gene knockout mice [52]. Furthermore, a positive correlation of Fetuin-A expression level in the liver with the expression of key glucose metabolism enzymes phosphoenolpyruvate kinase 1 and glucose-6-phosphatase has been reported [12,162], suggesting its fundamental role in insulin signaling and energy metabolism. Subsequent studies further demonstrated that circulating Fetuin-A protein levels were increased in NAFLD patients with insulin resistance and obesity, independent of adiposity [162,163,164,165]. In addition, several studies provided valuable insights for understanding Fetuin-A’s mechanism of action in the induction of insulin resistance, with Fetuin-A provoking the inflammatory response by increasing pro-inflammatory cytokine production in monocytes and adipocytes [53]. Furthermore, Fetuin-A serves as an endogenous ligand for toll-like receptor 4, mediating free fatty acid-inflicted TLR4 signaling activation to induce insulin resistance (Figure 2) [12,15,54,55]. In line with this notion, Fetuin-A was found to negatively regulate adiponectin production, thus affecting systemic insulin resistance [15,166,167]. Based on its role in metabolic processes, numerous pharmacological intervention studies for Fetuin-A have been examined in the metabolic disease model and it was found that liraglutide and pioglitazone reduce the circulating levels of Fetuin-A, while salsalate decreases Fetuin-A mRNA levels [115,168,169,170]. Collectively, given the significant contribution of Fetuin-A in inflammatory signaling and metabolic processes, it might represent a potential target for the treatment of metabolic diseases, including insulin resistance and type 2 diabetes.

Fetuin-B is another member of the Fetuin family, which shares ~24% of sequence homology with Fetuin-A in rodents and humans [157,161]. Similar to Fetuin-A, Fetuin-B is primarily produced and secreted by the liver, with its expression also detected in other tissues, yet to a lesser extent [157,161]. Recent cell-based studies revealed that Fetuin-B induces insulin resistance in cultured hepatocytes, as well as myotubes [26] and promotes lipid accumulation in HepG2 cells presumably via its capacity for decreasing AMPK activity, while activating liver-X-receptor α-SREBP-1c signaling (Figure 2) [56]. In line with these observations, Fetuin-B administration to lean mice resulted in glucose intolerance, with a mild effect on insulin resistance [26], while its liver-specific knockdown improved glucose tolerance in mice [26], in addition to lowering TG levels in the liver and plasma [56]. Emerging evidence from human studies has also demonstrated that circulating Fetuin-B levels are upregulated in patients with NAFLD, which are closely associated with insulin resistance [26,171]. Furthermore, the plasma concentration of Fetuin-B significantly correlated with clinical indices of obesity, hepatic steatosis, and homeostatic model assessment for insulin resistance [172]. Given their close relationship, the plasma levels of both Fetuin-A and -B were elevated and exhibited an inverse correlation with liver fibrosis stage in NAFLD patients [173,174]. However, when the association between Fetuin-A and -B was investigated in two independent cohorts, it was described that they may regulate glucose metabolism in slightly different manners, as Fetuin-A seems to modulate insulin signaling, while Fetuin-B may affect glucose effectiveness [175], suggesting that more comprehensive approaches are required to define how Fetuin-B regulates these metabolic processes in concert with Fetuin-A. Nevertheless, based on the findings from a number of studies, Fetuin-B is another potential therapeutic target in the treatment of metabolic diseases, such as obesity and insulin resistance.

### 4.3. Fibroblast Growth Factor 21 (FGF21)

Based on numerous studies that demonstrate its broad and significant impact on systemic energy metabolism in response to various metabolic challenges, hepatokine *FGF21* holds promise as a potential therapeutic for a wide range of metabolic abnormalities, including obesity and type 2 diabetes [12,13,14,15,176]. FGF21 is expressed in various organs, including the liver, white and brown adipose tissue, as well as the pancreas [57,176], but circulating FGF21 is predominantly derived from the liver [177]. Accumulating studies have demonstrated that the plasma levels of FGF21 increase in parallel with the severity of hepatic steatosis in humans [176,178], and are positively associated with TG, fasting insulin, and other insulin resistance indices, while negatively correlated with body mass index and HDL [179,180]. In agreement with this notion, serum FGF21 levels were positively correlated with the steatosis grade and NAFLD activity score in obese children and patients with advanced NASH, respectively [30,181,182]. Taken together, these results strongly suggest that the circulating FGF21 level may reflect the severity of hepatic steatosis and may indicate the early onset of NAFLD progression, thus representing a candidate biomarker of metabolic disorders [12,180]. Interestingly, emerging evidence also suggests that FGF21 positively regulates a variety of cellular processes and improves various metabolic abnormalities, as it markedly promotes glucose uptake in cultured adipocytes [57], enhances pancreatic β cell function and insulin secretion [58], upregulates fatty acid oxidation and insulin sensitivity, and improves hepatic steatosis and glycemic control [58,59,60]. The beneficial roles of FGF21 on metabolism are further validated by several studies using animal models deficient for FGF21 or its cognate receptor, which exhibited increased adiposity, exacerbated hepatic fat accumulation, insulin resistance, and hyperglycemia [14,183,184], suggesting that circulating FGF21 may be increased in patients with NAFLD in order to maintain energy homeostasis under metabolic stress. Of note, FGF21 levels are regulated by various physiological stimuli, such as fasting and exercise. As a representative fasting hormone, the expression of FGF21 is increased following prolonged fasting through the induction of PPARα and mediates its beneficial metabolic effects (Figure 2) [185,186]. In addition, physical training upregulated serum FGF21 levels in humans and rodents, in conjunction with increased *FGF21* gene expression in skeletal muscle and liver, as well as enhanced effects of FGF21 in adipose tissue [187,188,189]. Given the promising therapeutic efficacy against obesity and type 2 diabetes in preclinical studies, several FGF21 analogues and mimetics have been developed as pharmacological intervention strategies and are currently undergoing several clinical trials in patients with obesity, type 2 diabetes, and NASH [115,190,191,192,193]. To date, these FGF21 analogues have shown substantial improvement in dyslipidemia, while exerting a marginal effect on glycemic control in patients with obesity and type 2 diabetes [115,190,191,192]. Additionally, these pharmacological interventions ameliorated hepatic steatosis, as well as several biomarkers of liver fibrosis, although the beneficial effects on histology and other clinical outcomes of NASH were not satisfactory [190,191,193], suggesting that more comprehensive and targeted approaches are required to improve the therapeutic efficacy and safety of FGF21 analogues.

### 4.4. Selenoprotein P

Selenoprotein P was initially identified as a carrier protein containing 10 selenocysteine residues, which are responsible for transporting selenium from the liver to extrahepatic tissues, including the brain and testes [194,195]. Selenoprotein P is a glycoprotein primarily secreted by the liver, and its expression is increased in humans with NAFLD, type 2 diabetes, and cardiovascular diseases, in close association with insulin resistance and hyperlipidemia [61,196,197]. In line with this notion, hepatic selenoprotein P expression is upregulated in animal models of diet-induced obesity and type 2 diabetes [14,61,197]. Moreover, circulating selenoprotein P levels are negatively correlated with adiponectin levels in type 2 diabetes patients, in agreement with observations from selenoprotein P-deficient mice [198], suggesting its involvement in signaling crosstalk with other organokines. Selenoprotein P treatment of cultured primary hepatocytes or immortalized myocytes inhibited insulin signaling, as indicated by the diminished insulin-stimulated phosphorylation of the insulin receptor and Akt, which led to an increase in hepatic glucose production and a decrease in glucose uptake by myotubes [61]. Likewise, in vivo administration of selenoprotein P led to hepatic and peripheral insulin resistance, while liver-specific genetic ablation significantly ameliorated systemic glucose intolerance in association with enhanced insulin signaling in the liver and skeletal muscle [12,61,62,63,64]. Collectively, most of the studies on the role of selenoprotein P in the progression of insulin resistance and type 2 diabetes strongly support its potential as a novel therapeutic target in various metabolic disorders.

### 4.5. Leukocyte Cell-Derived Chemotaxin 2 (LECT2)

LECT2 is a liver-secreted protein that was initially identified as a neutrophil chemotactic factor promoting the growth of osteoblasts and chondrocytes [199]. LECT2 is primarily expressed and secreted from the liver, and, to a lesser extent, from adipose tissue, neurons, as well as white blood cells [15]. LECT2 is highly sensitive to changes in dietary fat content, as well as to the severity of hepatic steatosis. For example, LECT2 serum levels are rapidly increased prior to body weight gain [200] and exhibit a positive correlation with the extent of NAFLD, insulin resistance, hepatic inflammation, and liver fibrosis [65,200,201,202,203]. Moreover, HFD feeding increased circulating and hepatic LECT2 levels in mice and humans [65,189], while the opposite result was obtained in mice following exercise training or pharmacological inhibition of dipeptidyl peptidase-4, as mediated via augmented AMPK phosphorylation [65,204]. Consistently, recent studies have also demonstrated less M1-type macrophages, as well as a lower M1/M2 ratio in the livers of LECT2-deficient mice, in close association with reduced hepatic inflammation (Figure 2) [66], implicating that increased LECT2 levels may be an early manifestation of NAFLD progression. In line with this notion, LECT2-deficient mice fed an HFD exhibited improved insulin sensitivity in skeletal muscle, as opposed to the mice administered recombinant LECT2 protein, which developed insulin resistance in skeletal muscle [65]. Interestingly, the loss of LECT2 did not affect insulin sensitivity in liver and adipose tissue [65], while LECT2 treatment impaired insulin signaling in both cultured myotubes and differentiated 3T3-L1 cells by inhibiting Akt phosphorylation [65,205], thus implying the existence of a complex, unknown regulatory mechanism of LECT2 in vivo.

### 4.6. Follistatin

Follistatin is a secretory glycoprotein that is expressed in almost every tissue throughout the body, with the liver serving as the primary organ responsible for the production of circulating follistatin [206]. Follistatin is a member of the TGFβ family and was initially identified as an inhibitor of follicle-stimulating hormone production in the pituitary gland [14,67]. Subsequent evidence revealed its myostatin-antagonizing effect, which suppresses skeletal muscle growth [14,15]. Recently, follistatin has drawn significant attention due to its possible association with energy metabolism. The serum follistatin level is increased in patients with NAFLD and type 2 diabetes [207,208], while individuals with reduced body weight after bariatric surgery exhibited a decrease in serum follistatin level, in conjunction with improved insulin sensitivity and glycemic control [68,209]. Interestingly, circulating follistatin levels are also controlled by other physiological factors, including prolonged fasting and/or exercise, both of which are characterized by a high glucagon-to-insulin ratio due to the increased energy demand [210]. Nevertheless, the physiological role of circulating follistatin remains to be fully characterized, as existing evidence from studies of genetically manipulated animals has been somewhat confusing. Mice with liver-specific overexpression of follistatin exhibited increased hepatic glucose production, as well as aggravated insulin resistance in adipose tissue and skeletal muscle accompanying whole-body glucose intolerance, while follistatin knockdown improved insulin sensitivity [14,15,68,69]. Therefore, follistatin may contribute to metabolic disease progression. On the other hand, it has been reported that increased follistatin levels following exercise promote glucose and free fatty acid uptake in skeletal muscle, inducing the differentiation of brown adipocytes [70]. Furthermore, follistatin promoted thermogenesis by upregulating the uncoupling protein 1 expression in both brown and white adipose tissues [70,71]. Collectively, given the promising role of follistatin in a broad range of biological processes, including energy metabolism, a more comprehensive investigation is necessary to clearly understand its physiological role and therapeutic potential in metabolic disease.

### 4.7. Hepassocin

Hepassocin, also known as hepatocyte-derived fibrinogen-related protein 1, is upregulated during liver regeneration and is frequently downregulated in hepatocellular carcinoma [211,212]. Its expression is transcriptionally regulated by hepatocyte nuclear factor-1α [212]. The therapeutic effect of recombinant human hepassocin has been reported for fulminant hepatic failure [213]. Hepassocin enhances the proliferation of hepatocytes in vitro and in vivo, reducing hepatocyte apoptosis. Furthermore, the administration of recombinant human hepassocin successfully improved survival in rats with chemically-induced fulminant hepatic failure [213].

Hepassocin contributes to insulin resistance and type 2 diabetes. Plasma hepassocin levels were increased in participants with impaired fasting glucose, impaired glucose tolerance, and newly diagnosed diabetes, when compared to those with normal glucose tolerance [72]. High glucose levels promote hepassocin expression via the STAT3 and PP2A-HNF1 pathways [214]. In patients with hyperglycemic crisis or streptozotocin-induced hyperglycemic mice, the increase in blood or hepatic levels of hepassocin was diminished after the hyperglycemia treatment [214]. Similar results were obtained in HFD-fed mice following the metformin or rosiglitazone treatment [72]. Both the administration of recombinant hepassocin and its hepatic overexpression promoted insulin resistance in mice, whereas hepassocin knockdown had the opposite effect [72]. Mechanistically, ERK1/2 was shown to mediate hepassocin-induced insulin resistance [185]. Another research group also demonstrated that the recombinant hepassocin treatment impaired insulin sensitivity in differentiated C2C12 cells through JNK activation and the suppression of basal AMPK phosphorylation [73]. These results support a role for hepassocin in the development of insulin resistance.

Serum hepassocin levels are elevated in subjects with NAFLD compared to those without NAFLD [74]. The treatment of hepatocytes with palmitate upregulated hepassocin expression through CCAAT/enhancer-binding protein β (C/EBPβ). Overexpression of hepassocin in hepatocytes upregulated lipogenic proteins, such as fatty acid synthase, acetyl-CoA carboxylase, and mature SREBP-1, in an ERK1/2-dependent manner, thereby increasing hepatic TG accumulation [188]. In addition, liver injury and inflammatory cytokines, such as interleukin-1β (IL-1β), IL-6, and tumor necrosis factor-α (TNFα), were induced via the hepatic overexpression of hepassocin. Furthermore, the scores for all the histological features comprising the NAFLD activity score (i.e., steatosis, hepatocyte ballooning, and lobular inflammation) were higher in hepassocin-transgenic mice than control GFP-transgenic mice. In contrast, the hepatic knockdown of hepassocin attenuated HFD-induced NAFLD in mice [188]. Taken together, hepassocin plays an important role in hepatic steatosis.

Obesity is closely associated with insulin resistance and NAFLD. Hepassocin levels were significantly upregulated in overweight and obese subjects [215]. Moreover, serum hepassocin was positively correlated with obesity indices, including body mass index, waist circumference, and fat areas (total, visceral, and subcutaneous) in humans [75]. Overexpression of hepassocin in epididymal adipose tissue increased fat weight and the size of adipocytes along with the induction of fatty acid synthase and SREBP-1. Hepassocin enhanced adipogenesis through an ERK1/2-C/EBPβ-dependent pathway [190]. These studies indicate that hepassocin is not only a useful biomarker candidate for obesity and obesity-related metabolic diseases, but that hepassocin also contributes to adipogenesis, insulin resistance, and hepatic steatosis.

### 4.8. Retinol Binding Protein 4 (RBP4)

Liver is the major organ, where most of the vitamin A in our body is stored in the form of retinyl esters. The liver enzyme hydrolyzes the retinyl ester into retinol, which in turn binds to the member of lipocalin family, RBP4 in the hepatocytes. The primary function of RBP4 is to transport retinol into the circulation [216]. Two RBP4 receptors have been identified, which are responsible for the retinol uptake across the cell membrane [216]. RBP4 also plays an important role in metabolic syndrome. Yang et al. showed that RBP4 promotes insulin resistance in obesity and type 2 diabetes [79]. The insulin tolerance test was evaluated in mice expressing human RBP4 in muscle and mice injected chronically with purified RBP4. Both of the in vivo experiments showed that the elevated serum RBP4 caused insulin resistance. In contrast, insulin action was improved in RBP4 knockout mice or mice treated with fenretinide, a synthetic retinoid with a bulky side chain, which causes renal excretion of RBP4 and lowering serum RBP4 levels. The RBP4 treatment increased PEPCK and glucose production in hepatocytes [79]. RBP4 directly promoted basal lipolysis in adipocytes [76] and also triggered adipose tissue inflammation by increasing adipose tissue macrophage and CD4 T cell infiltration, resulting in systemic insulin resistance [81]. Hepatocytes are the major source of circulating RBP4 [217], but adipocytes also secrete it. Adipocyte-specific overexpression of RBP4 also increased hepatic gluconeogenic gene expression. In addition, it promoted the hepatic uptake of adipose-derived circulating free fatty acids and de novo lipogenesis, and suppressed hepatic free fatty acid oxidation [218]. The RBP4 overexpression contributes to hepatic LCAD hyperacetylation through the suppression of SIRT3 activity [78]. The study using RBP4 transgenic mice also demonstrated that RBP4 stimulated hepatic mitochondrial dysfunction and induced hepatic steatosis [78]. However, a model for liver-specific RBP4 overexpression in mice, established by adeno-associated viruses that express a highly liver-specific promoter (LP1)-RBP4, showed that liver-specific RBP4 overexpression did not induce glucose intolerance and had no effect on energy metabolism [82]. These contradictory results implicate that the tissue-specific RBP4 may have distinct roles in the development of insulin resistance.

Serum and adipose tissue RBP4 expression is subsequently increased in the obese subjects [76], whereas serum RBP4 levels are decreased with exercise and bariatric surgery [77,219]. In addition, those levels are elevated in patients with NAFLD, insulin resistance or type 2 diabetes [220,221,222]. Clinical evidence suggests that RBP4 is associated with hepatic steatosis and insulin resistance.

### 4.9. SPARC-Related Modular Calcium-Binding Protein 1 (SMOC1)

SMOC1 belongs to the BM-40 family, which is characterized by an extracellular calcium-binding domain and follistatin-like domain [223]. SMOC1 is a glycoprotein, which exhibits a widespread expression in various tissues [224]. It regulates embryonic development and pathophysiological processes, including osteoblast differentiation, ocular and limb development, angiogenesis, aortic valve calcification, and thrombin activation [225,226,227,228,229,230]. Montgomery et al. demonstrated that SMOC1 is a glucose-responsive hepatokine [83]. SMOC1 attenuated cAMP-PKA signaling and hepatic glucose output, and thereby improved glycemic control in lean, obese pre-diabetic, and diabetic *db/db* mice. Daily injection of SMOC1 recombinant protein for 3 weeks failed to show long-lasting metabolic improvement due to its rapid clearance, but a SMOC1-Fc fusion protein (fused to human immunoglobulin G1 Fc) exhibited the increased stability and therapeutic potential. Interestingly, the SMOC1-Fc treatment improved glycemic control, even better than the first-line diabetes medication metformin. However, it did not change the body weight and food intake. Plasma SMOC1 levels are downregulated in obese, insulin-resistant humans, and its levels are positively correlated with the glucose infusion rate during the hyperinsulinemic-euglycemic clamp and hepatic insulin sensitivity [83]. These results indicate that SMOC1 can be a promising therapeutic strategy in patients with type 2 diabetes.

### 4.10. Growth Differentiation Factor 15 (GDF15)

GDF15, a member of the TGF-β superfamily, is the ligand for the GFRAL receptor, which is abundantly expressed in neurons of the area postrema and nucleus of the solitary tract in mice and humans [89,231]. When GDF15 binds to GFRAL, it forms a complex with a co-receptor, RET. Then, RET is phosphorylated, which leads to downstream signaling via AKT, ERK1/2, and phospholipase C-γ [87]. Several inflammatory cytokines, such as IL-1β, IL-2, and TNF-α, increase GDF15 expression, and thereby circulating GDF15 levels are elevated in diabetes and cardiovascular diseases [15,232]. Circulating GDF15 levels are also increased in response to metformin, colchicine, AICAR, and cisplatin [233]. The increasing serum GDF15 expression is associated with weight loss in patients with advanced prostate cancer [234]. GDF15 has potent anti-obesity actions [88]. GDF15 increased thermogenesis, lipolysis, and oxidative metabolism [85]. The treatment of human GDF15 decreased food intake, resulted in weight loss, and improved insulin sensitivity, which was due to the increased oxidative metabolism and lipolysis [86,235]. Transgenic mice that ubiquitously overexpress the human GDF15 weighed less, although the food consumption was similar to wild-type mice [236]. Both the GDF15-treated mice and GDF15 transgenic mice exhibited less body weight compared to the respective controls. However, the effect of GDF15 administration and GDF15 transgenic mice on food intake was conflicting. A recent study from Borner et al. provided another evidence that GDF15 has an anorectic action through nausea and emesis [90]. GFRAL is essential for GDF15-induced food intake reduction and weight loss in mice [87]. GFRAL knockout mice exacerbated diet-induced obesity and insulin resistance [87]. These findings demonstrate that targeting GDF15-GFRAL may be an attractive therapeutic approach for obesity and comorbidities or eating disorders.

## 5. Conclusions and Perspectives

The global prevalence of NAFLD currently stands at approximately 25%, and its incidence is rapidly growing due to the obesity pandemic [237]. The liver as a key nutrient-sensing organ is dynamically exposed to changes in nutrient availability through the gut-liver axis or systemically and regulates the whole-body energy metabolism. This anatomical and physiological characteristic of the liver can partially explain why NAFLD progression frequently precedes other forms of metabolic diseases, such as type 2 diabetes and dyslipidemia. It is now widely appreciated that the impact of NAFLD on whole-body metabolic diseases is primarily attributed to the dysregulated secretome profile of steatotic hepatocytes following metabolic stress.

Hepatokine profiles are greatly affected by the metabolic status (Table 1). The liver communicates with other major metabolic organs to maintain the energy balance by producing and releasing hepatokines, which play an important role in this inter-organ communication network. Emerging evidence has now revealed that a number of hepatokines possess a metabolic capacity to regulate a myriad of biological processes in multiple extrahepatic tissues. In addition, hepatokines mediate the physiological benefit and/or influence in certain circumstances, such as exercise [238] and fasting/refeeding transition [239,240], suggesting that hepatokines can be attractive and important targets for the maintenance of metabolic homeostasis. Hepatokine dysregulation is implicated in the development of NAFLD and insulin resistance. Given the molecular features and metabolic functions of hepatokines, it is highly likely that both the chronic liver disease (namely NAFLD) and metabolic disturbances at the systemic level can be concurrently ameliorated by targeting candidate hepatokine(s) that exhibit autocrine, paracrine, and endocrine roles.

Currently, a liver biopsy is the gold standard for NAFLD and NASH diagnosis. However, several limitations, including the risk of bleeding or infection have restricted its widespread utilization, suggestive of the necessity for the development of reliable and valid non-invasive biomarkers. In NAFLD, the secretion of several hepatokines, such as Fetuin-A, Fetuin-B, FGF21, selenoprotein P, LECT2, follistatin, and hepassocin, is upregulated. This holds promise for the development of multi-hepatokine biomarker panels for NAFLD and NASH. The identification and evaluation of hepatokines, that are dynamically and specifically modulated under various physiological statuses, may be of help in developing new biomarkers, since this would provide more insights in deciphering the chronic disease progression stage and thus, developing the personalized therapy strategies that have multiple metabolic complications (e.g., metabolic syndrome).

Collectively, we summarized and discussed the role of hepatokines in the regulation of lipid and glucose metabolism at molecular, cellular, and systemic levels. In vitro and in vivo studies have shown that hepatokine inhibition, overexpression or recombinant hepatokine treatment have a major influence on NAFLD and/or insulin resistance. Based on these preclinical findings, hepatokine mimetics or inhibitors may represent effective therapeutics for NAFLD and other metabolic diseases.

## Figures and Tables

**Table 1:** Target organs or cells of hepatokines and their biological functions.

Hepatokines	Target Organs or Cells	Biological Functions	Reference
ANGPTL3	WAT, muscle, liver	Suppressed LPL and endothelial lipase Increased plasma TG and FFA Increased VLDL-TG secretion (liver) Increased uptake of VLDL-TGs (WAT) Decreased glucose uptake (WAT) Promoted lipogenesis and inflammatory response (liver)	[15,27,28,29,30,31,32,33]
ANGPTL4	WAT, vascular endothelial cells	Inhibited LPL activity Increased plasma TG levels NAFLDIncreased adipocyte lipolysis Suppressed hepatic glucose production	[15,34,35,36,37,38,39]
ANGPTL6	skeletal muscle, WAT, liver	Enhanced insulin signaling (skeletal muscle) Inhibited gluconeogenic pathway (liver) Increased mitochondrial oxygen consumption (WAT)	[40,41,42,43]
ANGPTL8	hepatocytes, adipocytes	Improved insulin signaling and suppressed gluconeogenic gene expression (liver) Suppressed lipolysis (hepatocyte, adipocyte) Promoted lipogenesis (liver)	[44,45,46,47,48]
Fetuin-A	liver, WAT, skeletal muscle, monocytes	Blocked insulin signaling through inhibition of insulin receptor tyrosine kinase (liver, WAT, skeletal muscle) Provoked inflammatory response (monocytes, adipocytes) Inhibited adiponectin production	[13,15,49,50,51,52,53,54,55]
Fetuin-B	hepatocytes, myotubes	Induced insulin resistance (hepatocytes, myotubes) Promoted lipogenesis (hepatocytes)	[26,56]
FGF21	WAT/BAT, liver, skeletal muscle, pancreas, CNS	Promoted glucose uptake (adipocytes) Stimulated thermogenesis (BAT) Enhanced insulin secretion (pancreatic β cells) Increased fatty acid oxidation and insulin sensitivity (liver, skeletal muscle) Reduced NAFLD Decreased VLDL uptake and lipogenesis (liver) Decreased alcohol and sugar intake Increased energy expenditure and decreased body weight (CNS)	[57,58,59,60]
Selenoprotein P	liver, skeletal muscle	Inhibited hepatic glucose production Decreased glucose uptake (skeletal muscle)	[12,61,62,63,64]
LECT2	liver, skeletal muscle	Increased M1/M2 ratio and hepatic inflammation (liver) Development of insulin resistance (skeletal muscle) Promoted lipid accumulation (liver)	[65,66]
Follistatin	pituitary, skeletal muscle, liver, skeletal muscle, WAT, BAT	Inhibition of FSH production (pituitary) Suppressed skeletal muscle growth via antagonizing myostatin Promoted insulin resistance (liver, skeletal muscle, WAT) Increased glucose and FFA uptake after exercise training (skeletal muscle) Induced differentiation of brown adipocytes Promoted thermogenesis (BAT)	[14,15,67,68,69,70,71]
Hepassocin	liver, skeletal muscle, WAT	Promoted insulin resistance NAFLD Adipogenesis (WAT)	[72,73,74,75]
RBP4	various peripheral tissues including retina	Increased lipolysis in adipocytes Promoted hepatic mitochondrial dysfunction and hepatic steatosisSerum RBP4 levels were associated with insulin resistance and components of metabolic syndrome in humans Depending on the source of RBP4 (hepatocytes or adipocytes), the effect of RBP4 is controversial. - RBP4 treatment increased PEPCK (liver) and impaired insulin signaling (muscle and adipocytes). - No effect of liver-secreted RBP4 on glucose homeostasis in mice	[76,77,78,79,80,81,82]
SMOC1	liver, skeletal muscle, etc.	Improved glycemic control via inhibiting gluconeogenesis and glucose output (liver)	[83]
GDF15	adipose tissue, skeletal muscle, liver, brain, heart, kidney	Anorexia Increased energy metabolism (liver, muscle, adipose tissue) and lowered body weight Stimulated thermogenic and lipolytic genes (BAT, WAT) Improved glucose tolerance and insulin sensitivity Prevented liver steatosis in HFD-fed mice	[84,85,86,87,88,89,90]

ANGPTL: Antiopoietin-like proteins; BAT: Brown adipose tissue; CNS: Central nervous system; FFA: Free fatty acid; FSH: Follicle-stimulating hormone; GDF15: Growth differentiation factor 15; HFD: High fat diet; LECT2: Leukocyte cell-derived chemotaxin 2; LPL: Lipoprotein lipase; RBP4: Retinol binding protein 4; SMOC1: SPARC-related modular calcium-binding protein 1; TG: Triglyceride; VLDL: Very low-density lipoprotein; WAT: White adipose tissue.
